# Systematic characterization of the peroxidase gene family provides new insights into fungal pathogenicity in *Magnaporthe oryzae*

**DOI:** 10.1038/srep11831

**Published:** 2015-07-02

**Authors:** Albely Afifa Mir, Sook-Young Park, Md. Abu Sadat, Seongbeom Kim, Jaeyoung Choi, Junhyun Jeon, Yong-Hwan Lee

**Affiliations:** 1 Department of Agricultural Biotechnology, Fungal Bioinformatics Laboratory, Center for Fungal Genetic Resources, and Center for Fungal Pathogenesis, Seoul National University, Seoul 151-921, Korea

## Abstract

Fungal pathogens have evolved antioxidant defense against reactive oxygen species produced as a part of host innate immunity. Recent studies proposed peroxidases as components of antioxidant defense system. However, the role of fungal peroxidases during interaction with host plants has not been explored at the genomic level. Here, we systematically identified peroxidase genes and analyzed their impact on fungal pathogenesis in a model plant pathogenic fungus, *Magnaporthe oryzae*. Phylogeny reconstruction placed 27 putative peroxidase genes into 15 clades. Expression profiles showed that majority of them are responsive to *in planta* condition and *in vitro* H_2_O_2_. Our analysis of individual deletion mutants for seven selected genes including *MoPRX1* revealed that these genes contribute to fungal development and/or pathogenesis. We identified significant and positive correlations among sensitivity to H_2_O_2_, peroxidase activity and fungal pathogenicity. In-depth analysis of *MoPRX1* demonstrated that it is a functional ortholog of thioredoxin peroxidase in *Saccharomyces cerevisiae* and is required for detoxification of the oxidative burst within host cells. Transcriptional profiling of other peroxidases in Δ*Moprx1* suggested interwoven nature of the peroxidase-mediated antioxidant defense system. The results from this study provide insight into the infection strategy built on evolutionarily conserved peroxidases in the rice blast fungus.

In plant-pathogen interactions, one of the first defense responses in plants is rapid and transient production of reactive oxygen species (ROS)^1^,^2^,^3^. ROS generation is spatially and temporally regulated. ROS are typically produced in the apoplastic compartment within minutes following initiation of pathogenic infection^4^,^5^,^6^,^7^. ROS and intracellular redox changes can be used by pathogenic fungi for signalling purposes^8^ as well as development and pathogenicity^9^,^10^. Although O_2_^–^ is the proximal product generated, the more stable H_2_O_2_ is the most abundant ROS. ROS can directly kill pathogens, produce cross-linked cell wall polymers to fortify physical barriers to pathogen entry, or act as a messenger in a cell signaling pathway, leading to pathogenesis-related (PR) gene expression and localized hypersensitive responses^1^,^3^,^11^,^12^,^13^,^14^.

Pathogens must effectively incapacitate production of host-driven ROS or detoxify ROS for successful infection of host cells^15^,^16^. Studies have demonstrated that H_2_O_2_ detoxification is an essential virulence determinant in fungal pathogens such as *Candida albicans*^17^, *Ustilago maydis*^18^, *Alternaria alternata*^19^,^20^, and *Magnaporthe oryzae*^18^,^21^,^22^,^23^. Furthermore, recent studies have identified a group of ROS-scavenging enzymes, peroxidases, as workhorses for the fungal antioxidant defense system^24^,^25^,^26^. Peroxidases (EC1.11.1.x) are enzymes that mediate electron transfer from H_2_O_2_ and organic peroxide to various electron acceptors. They are evolutionarily conserved and implicated in biological processes as diverse as immune responses and hormone regulation^27^,^28^,^29^,^30^. Examples of peroxidase enzymes include NAD(P)H oxidase, catalase, glutathione peroxidase, catalase peroxidase, ascorbate peroxidase, lignin peroxidase, and peroxiredoxin^31^.

Pathogenic fungi are becoming great threats to both plant and animal and are jeopardizing food security^32^. The hemibiotrophic fungus *M. oryzae* is a causal agent of the rice blast, one of the most devastating disease in cultivated rice^33^. This disease is estimated to destroy an amount of rice that would feed 60 millions of people annually^33^. The fungus is genetically tractable and can undergo infection-specific development in a laboratory setting. Since the full genome sequences of *M. oryzae* and rice are publicly available^34^, rice blast is a model system for studying plant-pathogen interactions.

The rice blast fungus experiences sequential developmental changes. The disseminated asexual spore, the conidium, attaches to the hydrophobic host surface upon hydration, and produces a cylindrical germ tube. The end of the germ tube forms a specialized dome-shaped infection structure called an appressorium. The mature heavily malanized appressorium mechanically penetrates the cuticular layer of host plants, using enormous turgor pressure (>8 Mpa)^33^,^35^. In a host plant, the fungus enters the host cytoplasm via a penetration peg, develops bulbous, infectious hyphae in the first-invaded plant cell, and, subsequently, grows into neighboring cells, presumably through the plasmodesmata^36^.

In *M. oryzae*, six peroxidase genes have been functionally characterized. Studies have provided evidence that both balancing the intracellular level of ROS and efficient removal of extracellular ROS are pivotal for early phase host infection. Genetic analysis of NADPH oxidase genes, *NOX1* and *NOX2*, suggests that regulation of intracellular ROS is required for appressorium maturation and penetration peg formation^37^. The deletion mutant of a glutathione peroxidase gene, *HYR1*, is less tolerant to ROS and produces smaller lesions on rice plants^24^. The role of other putative peroxidase genes in antioxidant defense is unknown.

Mutants lacking the secreted large subunit catalase, *CATB*, are less pathogenic due to their involvement in strengthening the fungal cell wall rather than detoxifying host-derived H_2_O_2_^38^. Deletion of the catalase peroxidase gene, *CPXB*, renders the mutant sensitive to exogenous H_2_O_2_, but it does not impair pathogenicity^39^. Finally, loss of the peroxiredoxin gene, *TPX1*, is required for pathogenicity, but not to neutralize plant-generated ROS^40^.

There is still much to learn about peroxidases and fungal pathogenicity. First, are specific peroxidases particularly important for pathogenicity? Second, what are the relationships among peroxidases, sensitivity to exogenous H_2_O_2_, and pathogenicity? In an attempt to answer these questions, we performed comprehensive expression and functional analyses based on reconstruction of phylogeny for 27 putative peroxidase genes in *M. oryzae*.

## Results

### Phylogenetic analysis of peroxidase genes in *M. oryzae*

For systematic analysis of peroxidase genes in *M. oryzae*, we first investigated the evolutionary relationships among peroxidase genes in filamentous fungi by constructing a phylogenetic tree. Searching through the Fungal Peroxidase Database (fPoxDB; http://peroxidase.riceblast.snu.ac.kr/), a total of 896 peroxidase genes were identified from genomes of 35 species that included 29 fungi, 1 chromista, 3 metazoa, and 2 viridiplantae (Supplementary information [SI] Table S1). In *M. oryzae*, we found 27 putative peroxidase-encoding genes. Protein sizes ranged from 160 (MoPRX3) to 1171 (MoLDS2) amino acids (Supplementary Table S2). SignalP (http://www.cbs.dtu.dk/services/signalP/) predicted nine of them (*MoAPX1*, *MoAPX2*, *CPXB*, *MoLIP1*, *MoLIP2*, *MoHPX1*, *MoHPX2*, *MoHPX3* and *CATB*) to contain signal peptides (Supplementary Table S2). Using amino acid sequences of putative peroxidases, including 27 *M. oryzae* peroxidases, the phylogeny of these peroxidases was reconstructed. The resulting phylogenetic tree revealed that the fungal peroxidases are not explicitly divided into distinct subgroups, with most nodes associated with subgroup branching and supported by low bootstrap values (Supplementary Fig. S1). Peroxidases sharing domain architecture were clustered into clades.

**Figure 1:**
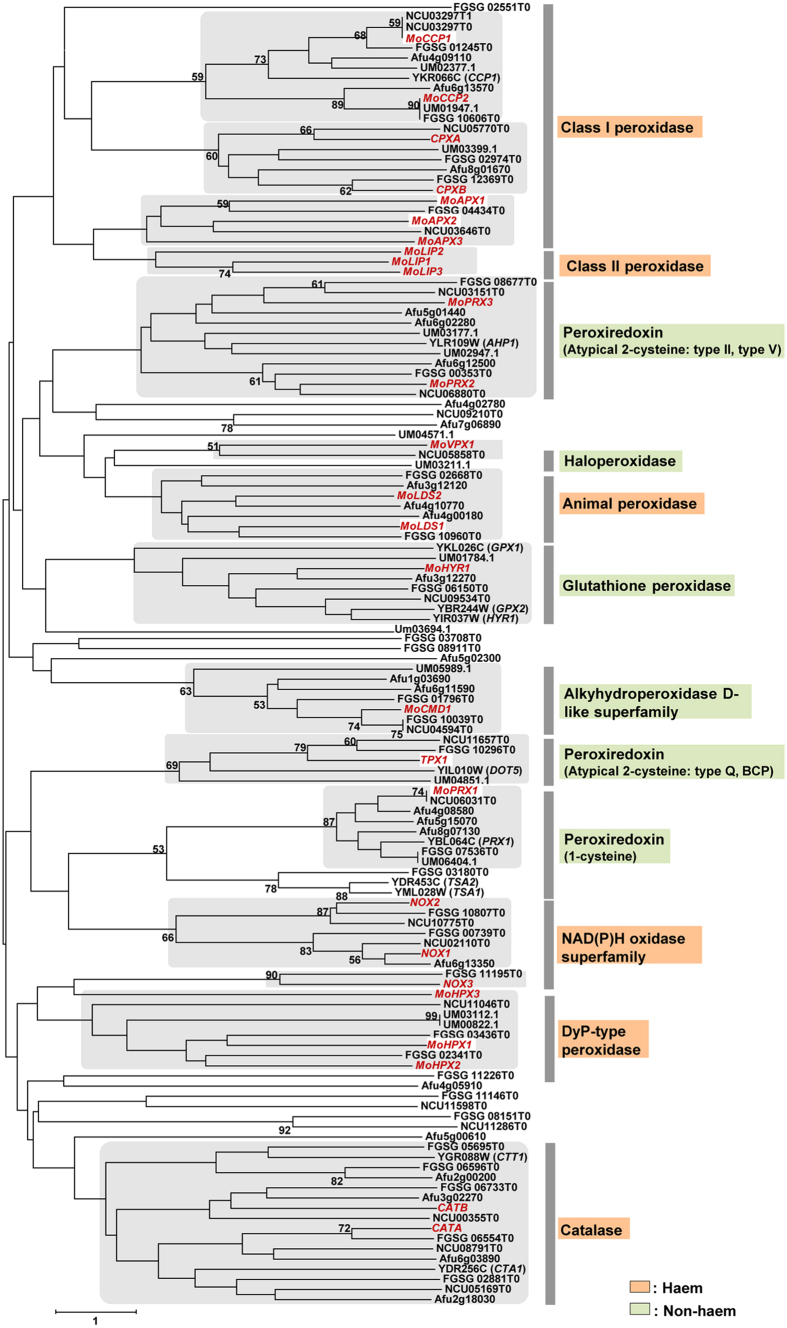
Phylogenetic analysis of putative peroxidase genes in six selected fungi. A neighbor-joining tree was constructed based on the amino acid sequences of representative fungal peroxidase genes. Numbers at nodes represent bootstrap confidence values, or percentage of clade occurrence in 2,000 bootstrap replicates; only nodes supported by >50% bootstraps are shown. The scale bar represents the number of amino acid differences per site. Sub-clades containing *Magnaporthe oryzae* peroxidase genes are shaded, in which *M. oryzae* peroxidase genes and characterized genes from other fungi are depicted in bold red or black, respectively. Abbreviations for fungal species, followed by their GenBank accession numbers, are as follows: Mo, *Magnaporthe oryzae*; Sc, *Saccharomyces cerevisiae*; Fg, *Fusarium graminearum*; Nc*, Neurospora crassa*; Um, *Ustilago maydis*; Af, *Alternaria fumigatus*.

To focus our analysis on fungal peroxidases, we selected six representative fungi that included three plant pathogenic fungi (*M. oryzae*, *Fusarium graminearum*, *Ustilago maydis*), one human pathogenic fungus (*Aspergillus fumigatus*), and two saprophytic fungi (*Neurospora crassa* and *Saccharomyces cerevisiae*). Phylogenetic analysis with selected fungal species placed 27 *M. oryzae* peroxidase genes into 15 clades (shaded box in Fig. 1). Among the 27 peroxidase genes in *M. oryzae*, 16 genes (*MoAPX3*, *CPXA*, *CPXB*, *MoCCP1*, *MoCCP2*, *NOX1*, *NOX2*, *CATB*, *CATA*, *MoLDS1*, *MoLDS2*, *MoPRX1*, *TPX1*, *MoPRX2*, *MoCMD1* and *HYR1*) were conserved in Ascomycota (Supplementary Table S3).

### Expression profiling of 27 *M. oryzae* peroxidase genes during fungal development and under oxidative stress

**Figure 2:**
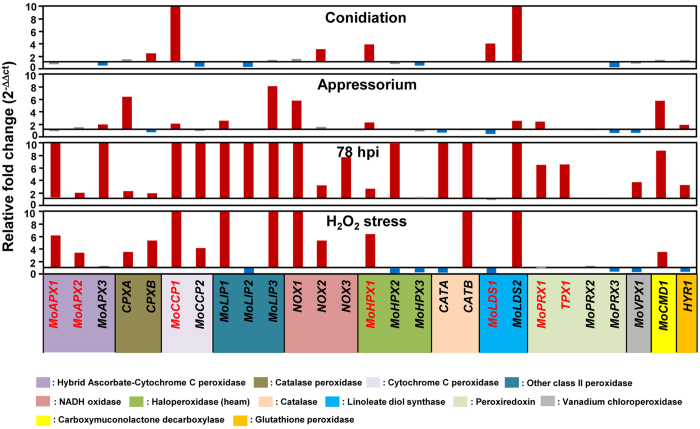
Expression profiling of 27 *M. oryzae* peroxidase genes during infection-related developmental stages and infection (78 hpi) on rice, and under oxidative stress. Upregulated genes (more than 1.5-fold) are indicated by red bars and downregulated genes (less than 0.5-fold) are denoted by blue bars. The genes not showing differential expression are marked in gray. Seven peroxidase genes were selected for functional analysis are highlighted as red.

As a next step, we performed expression profiling of the 27 peroxidase-encoding genes using qRT-PCR during infection-related developmental stages that included conidiation, appressorium formation, and 78 h post incubation (hpi) on rice plants and under oxidative stress with 2.5 mM H_2_O_2_ (Fig. 2). Expression analysis revealed that most of the peroxidase genes were differentially expressed under the imposed conditions. Compared to expression in mycelia, only a few genes were upregulated in developmental samples, including the conidia and appressoria. However, we found that a majority of the genes (23 genes or 85.2%) were upregulated during the infection stage at 78 hpi. Fourteen genes (51.9%), the exceptions being *MoAPX3, MoLIP2, NOX3, MoHPX2, CATA, MoPRX1, TPX1, MoVPX1 and HYR1*, were also upregulated under H_2_O_2_ stress conditions (Fig. 2). Such an expression pattern suggested the possibility of peroxidase genes playing roles associated with ROS during plant infection.

### Genetic analysis of peroxidase genes and fungal pathogenicity

Based on groupings from phylogenetic analysis, we prioritized seven peroxidase genes for functional analysis. We selected the genes from clades that did not contain previously characterized genes (Fig. 1). The seven genes included three genes (*MoAPX1*, *MoAPX2* and *MoCCP1*) from class I peroxidase, one gene (*MoHPX1*) from Dye-type peroxidase, one gene (*MoLDS1*) from animal peroxidase, and two genes (*MoPRX1* and *TPX1*) from peroxiredoxin peroxidase (Fig. 2 and Supplementary Table S1). Targeted gene disruption was conducted using the KJ201 strain as wild-type to genetically assess the impact of selected putative peroxidase genes on fungal development and pathogenicity. Knockout constructs were prepared using double-joint PCR and directly used for transformation of wild-type protoplasts. The resulting hygromycin-resistant colonies were screened by PCR, and the correct gene replacement event for each targeted gene was confirmed by Southern blot analysis (Supplementary Fig. S2).

**Figure 3:**
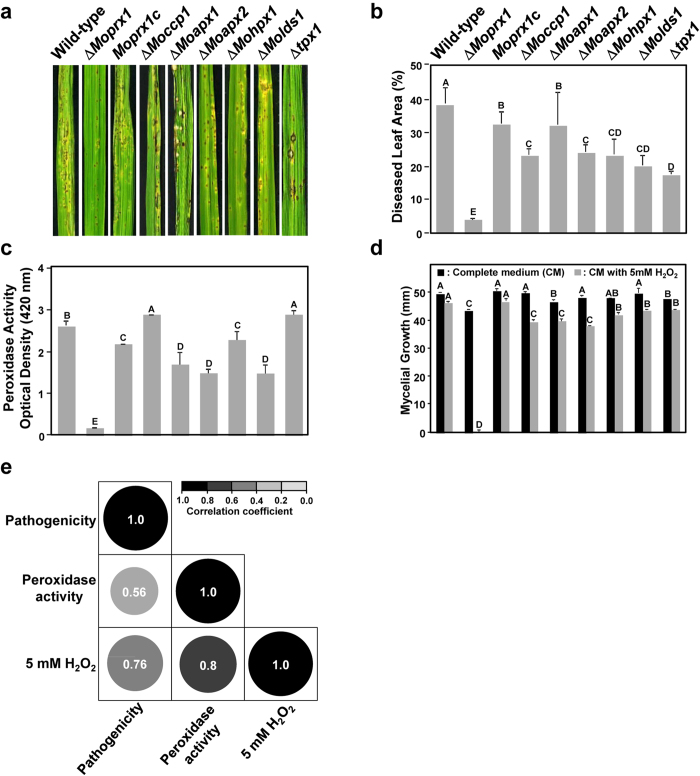
Pathogenicity, peroxidase activity and mycelial growth of the strains. (**a**) Disease symptoms on rice leaves, wild-type and seven peroxidase gene deletion mutants, including Δ*Moprx1*, Δ*Moccp1*, Δ*Moapx1*, Δ*Moapx2*, Δ*Mohpx1*, Δ*Molds1*, and Δ*tpx1*, and one complement strain *Moprx1c*. The diseased leaves were collected at 7 dpi. (**b**) Disease leaf area (DLA) percentage measured using the Image J software. (**c**) Extracellular peroxidase activity by ABTS oxidizing assay. (**d**) Mycelial growth of seven deletion mutants under oxidative stress. (**e**) Pearson correlation coefficients among DLA from pathogenicity tests, peroxidase activity, and mycelial growth on 5 mM H_2_O_2;_ data generated from the wild-type and seven peroxidase gene deletion mutants. Error bars represent SD of the mean of three independent experiments. Bars with the same letters are not significantly different (Duncan’s multiple comparisons test, *P* < *0.05*).

Deletion of seven genes, one gene at a time, had minor effects on vegetative growth (Fig. 3d), conidiation, conidial germination, and appressorium formation. The exception was the deletion of *TPX1*, which caused a substantial decrease in conidia production (Supplementary Table S4). When we spray-inoculated 3-week-old susceptible rice seedlings with spores from wild-type and deletion mutant strains (~5 × 10^4^ spores/ml), all seven deletion mutants (Δ*Moprx1*, Δ*Moccp1*, Δ*Moapx1*, Δ*Moapx2*, Δ*Mohpx1*, Δ*Molds1*, and Δ*tpx1)* showed reduced pathogenicity compared to the wild-type, in terms of diseased leaf area (DLA; Duncan’s multiple range test, P < *0.05*; Fig. 3a,b). In particular, we found that deletion of *MoPRX1* resulted in the most dramatic reduction in both number and size of lesions on rice leaves.

### Peroxidase activity, sensitivity to H_2_O_2_, and pathogenicity

The above observation that all the mutants were less pathogenic than the wild-type prompted us to explore the relationships among peroxidase activity, sensitivity to exogenous H_2_O_2_, and pathogenicity. Peroxidase activity was quantified by measuring absorbance of 2, 2´-azino-bis (3-ethylbenzthiazoline-6-sulphonate; ABTS) at 420 nm in culture filtrates of seven deletion mutants and the wild-type. In our assay for peroxidase activity, the deletion mutant strains Δ*Moprx1*, Δ*Moapx1*, Δ*Moapx2*, Δ*Mohpx1*, and Δ*Molds1* showed reduced peroxidase activity compared to the wild-type (Fig. 3c). In particular, the mutant Δ*Moprx1* lost almost all peroxidase activity. We then went on to assess the sensitivity of the deletion mutants to exogenous H_2_O_2_ (Fig. 3d). All seven mutants were sensitive to H_2_O_2_, suggesting that their ability to cope with oxidative stress was compromised to varying degrees. Again, Δ*Moprx1* showed hypersensitivity to the treatment of H_2_O_2_. Such dramatic phenotypic defects of the Δ*Moprx1* mutant could be complemented by reintroducing a functional copy of the *MoPRX1* gene (Fig. 3 and Supplementary Fig. S2).

When Pearson’s correlation coefficients were computed among pathogenicity (DLA), peroxidase activity, and sensitivity to H_2_O_2_; some of the coefficients were significant. The highest correlation was between peroxidase activity and sensitivity to H_2_O_2_ (r = 0.8). The next highest correlation was between pathogenicity and sensitivity to H_2_O_2_ (r = 0.76). The correlation between pathogenicity and peroxidase activity (r = 0.56) was relatively low (Fig. 3e).

### *MoPRX1* as a conserved peroxidase

**Figure 4:**
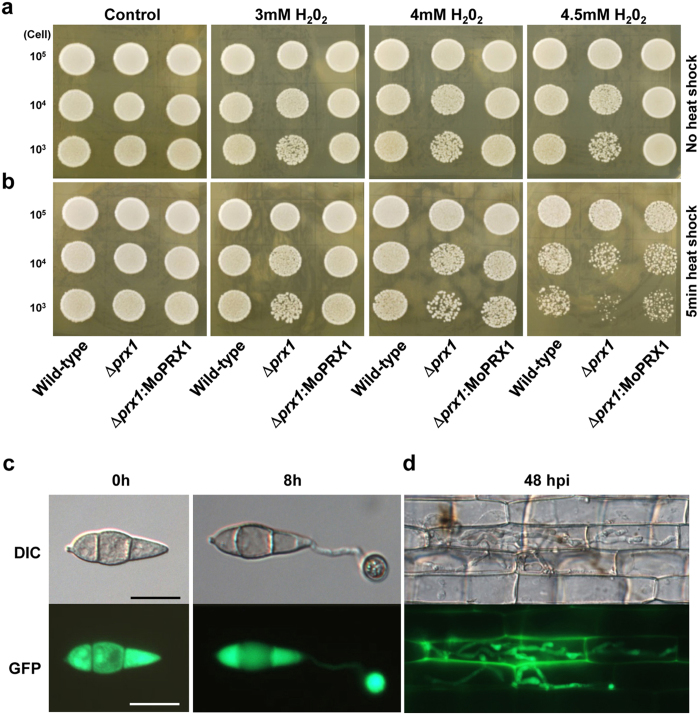
MoPRX1 complements *S. cerevisiae* PRX1 and localized in cytoplasm. For yeast complementation test, cells were cultured overnight, diluted in phosphate-buffered saline at a final concentration adjusted to 2 × 10^3^ cells/μl, and aliquoted in 100-μl samples for each strain. Diluted 10 μl aliquots (10 μl containing 10^3^, 10^4^, and 10^5^ cells/μl) of wild-type (BY4742), Δ*prx1* (YBL064C), and Δ*prx1*:*MoPRX1* were exposed to 3, 4 and 4.5 mM H_2_O_2_ on YPD agar plates after (**a**) with or (**b**) without 5-min heat shock at 50 °C. (**c**) Cellular localization of MoPRX1::GFP fusion protein in a conidium and appressoria of *M. oryzae*. (**d**) Infectious hypha expressing MoPRX1::GFP on rice sheaths at 48 hpi. DIC images were captured using a 20-ms exposure to transmitted light with a DIC filter. Fluorescence images were captured using a 400-ms exposure to absorbed light using a GFP filter. Bar = 10 μm.

In response to the general trend observed in our comprehensive approach, we investigated peroxidase-mediated fungal pathogenesis through an in-depth analysis of *MoPRX1*. MoPRX1 (224 aa) is predicted to contain two conserved domains, the thioredoxin fold domain (IPR012335) and the C-terminal peroxiredoxin domain (IPR09479). MoPRX1 is highly homologous to PRX1 in *S. cerevisiae*, which is a member of the peroxiredoxin family with 1-cysteine (proteins that contain 1 conserved cysteine directly involved in catalysis). We used a deletion mutant in the yeast gene *PRX1* to test the peroxidase activity of MoPRX1. PRX1 in *S. cerevisiae* is localized in mitochondria and is capable of removing organic hydroperoxides and H_2_O_2_, providing protection against oxidative stresses. Thus, the yeast mutant of *PRX1* is considerably less tolerant to H_2_O_2_ treatment and heat-shock is known to exacerbate this phenotype^41^. When a full-length cDNA of *MoPRX1* was introduced into a yeast strain lacking *PRX1* (YBL064c), the *MoPRX1* gene could restore the tolerance of the mutant to exogenous H_2_O_2_ to the wild-type level with or without heat-shock, indicating that *MoPRX1* is a functional ortholog of yeast *PRX1* (Fig. 4a,b).

Despite functional conservation, however, MoPRX1 was not localized to mitochondria in *M. oryzae*. When a fusion construct of the *MoPRX1* promoter-*MoPRX1*-GFP was prepared and introduced into ∆*Moprx1*, a strong fluorescent signal was observed in the cytoplasm of conidia and appressoria (Fig. 4c). The *PRX1* of a human pathogenic fungus, *Candida albicans*, was reported to translocate from the cytoplasm to the nucleus during hyphal transition. However, cytoplasmic localization of the protein was consistently observed, even in infectious hyphae of the rice blast fungus invading rice plant cells (Fig. 4c).

### Roles of *MoPRX1* during early phase of host infection

**Figure 5:**
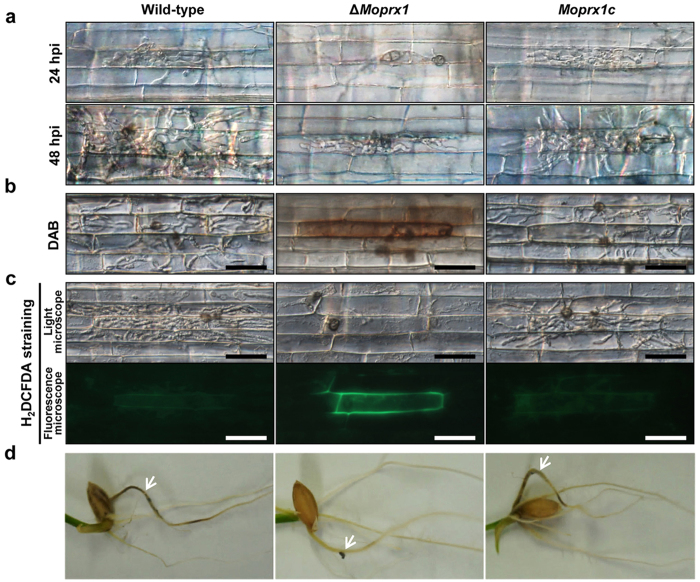
MoPRX1 detoxifies host ROS and is involved in rice root infection. (**a**) Sheath assays with compatible *Oryza sativa* L. cv. Nakdongbyeo showed delayed and restricted growth of Δ*MoPrx1* in rice cells. Sheaths were observed at 24 and 48 hpi inoculated with 2 × 10^4^ conidia/ml. (**b**) ROS detection by DAB (3,3´-diaminobenzidine) staining, and (**c**) H_2_DCFDA (5-(and-6)-chloromethyl-2′,7′-dichlorodihydrofluorescein diacetate acetyl ester) staining in inoculated rice sheaths at 48 hpi. DIC images were captured using a 20-ms exposure to transmitted light with a DIC filter. Fluorescence images were captured using a 200-ms exposure to absorbed light using a GFP filter. Bar = 50 μm. (**d**) Root infection from rice seedlings (*Oryza sativa* L. cv. Nakdongbyeo) by wild-type, Δ*Moprx1* and *MoPRX1c* strains. White arrow indicates the infection site on the root surface.

To elucidate the contribution of MoPRX1, as a peroxidase, to fungal pathogenicity, we monitored wild-type and Δ*Moprx1* during the early phase of host infection using the rice sheath assay. When rice sheath cells were inoculated with fungal strains, the infection process was severely delayed in Δ*Moprx1*. In contrast to the wild-type and complementation strain (*Moprx1*c), which penetrated and colonized the first-invaded cell at 24 hpi, the mutant barely elaborated a penetration peg. At 48 hpi, infectious growth of Δ*Moprx1* was mostly confined to one to two host cells, while that of the wild-type involved a larger number of cells (Fig. 5a). A similar pattern of delay in the early infection phase was observed in experiments using onion (*Allium cepa* L.) cells as a surrogate (Supplementary Fig. S3). Our observations identified a reduced number of small lesions from rice leaves spray-inoculated with Δ*Moprx1* (Fig. 3a).

One possible explanation for such delayed infection is that the mutant was inherently defective in the appressorium-mediated penetration process, including penetration peg formation. To probe this possibility, conidial suspensions from Δ*Moprx1*, *Moprx1*c, and the wild-type were infiltrated into rice leaves. This allowed the fungus to gain access to plant tissues without appressorium-mediated penetration. In an infiltration experiment, Δ*Moprx1* produced smaller disease lesions compared to the wild-type and *Moprx1*c, suggesting that *MoPRX1* plays roles in the post-penetration phase (Supplementary Fig. S3d).

Based on our results on the peroxidase activity and H_2_O_2_ sensitivity of Δ*Moprx1*, we hypothesized that *MoPRX1* is involved in coping with host-derived ROS during early infection. To test this, accumulated H_2_O_2_ at the infection sites was examined by 3, 3´-diaminobenzidine (DAB) staining at 48 hpi, using the sheath assay. The results showed that rice sheath cells infected by wild-type and *Moprx1*c were not stained by DAB, whereas the rice cells infected with Δ*Moprx1* were strongly stained, indicating a high concentration of H_2_O_2_ (Fig. 5b). We confirmed the accumulation of ROS using the dye 2´, 7´-dichlorodihydrofluorescein diacetate (H_2_DCFDA, Life technology D-399). H_2_DCFDA staining also showed fluorescence in Δ*Moprx1* infection sites, signifying ROS accumulation, but not in the wild-type or *Moprx1*c (Fig. 5c). This association of ROS accumulation with the fungus lacking *MoPRX1* suggests that *MoPRX1* is implicated in direct and/or indirect detoxification of ROS.

A previous study has shown that *M. oryzae* can infect root tissue, and that this infection requires the fungus to undergo various types of programmed development, which is typical of a root-infecting pathogen^42^. The authors of that study also identified a tissue-specific virulence factor, *MgFOW1*. Therefore, our goal was to determine if *MoPRX1* is required for root infection. Our pathogenicity assay using roots of rice seedlings showed that Δ*Moprx1* cannot cause disease symptoms on root tissues, in contrast to the wild-type causing root browning (Fig. 5d). This suggests that *MoPRX1* is a virulence factor required for infection of multiple tissues.

### Transcription of other peroxidase genes can be perturbed by deletion of *MoPRX1*

The observation that ROS accumulation substantially increases in the absence of *MoPRX1* led us to examine the expression of a further 26 peroxidase genes using qRT-PCR in the Δ*Moprx1* background, compared to the wild-type. We included in this experiment *MoPRX1* to confirm absence of its transcripts in the mutant strain. Expression of *MoAPX1*, *MoLIP3*, *NOX1*, *NOX2*, *MoHPX2, MoHPX3, MoLDS1, MoPRX2, and MoVPX1* was upregulated (>1.5-fold), whereas expression of *MoLIP1*, *MoLIP2*, *CATA*, *CATB* and *MoPRX3* was downregulated (<0.5-fold) as shown in supplementary Fig. S4. Expression of the remaining genes remained unchanged. No *MoPRX1* transcript was detected, as expected. CII peroxidases (*MoLIP1*, *MoLIP2*) and catalase (*CATB*), which are predicted to be secreted peroxidases having signal peptides (Supplementary Table S2), were downregulated in Δ*Moprx1* (Supplementary Fig. S4). Our results showed that deletion of *MoPRX1* can alter transcript levels of other peroxidase genes and thus extracellular peroxidase activity.

## Discussion

Successful infection of hosts requires pathogens to be equipped with means to breach their defense strategies^15^,^16^,^22^. The most prominent response of host plants to pathogen attack is a burst of ROS production. In response, pathogens have evolved antioxidant defense systems^43^. Recently, several studies have suggested that at least some of the peroxidases are integral components of such an antioxidant defense system in *M. oryzae*^24^,^37^,^38^,^39^,^40^. Searching of the Fungal Peroxidase Database (fPoxDB)^44^ revealed 27 putative peroxidase genes encoded in the genome of *M. oryzae*, leaving the general role of peroxidases in fungal pathogenesis open to question. We classified those 27 putative peroxidase genes based on our phylogenetic analysis, and conducted functional analysis of a subset of the 27 genes.

Our expression profiling of 27 peroxidase genes showed that the majority (23 genes or 85%) of peroxidase genes were induced in infected plant samples (78 hpi), and 14 genes were upregulated under H_2_O_2_ stress (Fig. 2). Seven peroxidase genes were upregulated during conidiation and 11 were upregulated during appressoria formation. Considering that the host environment involved high levels of ROS, these data suggest that transcription of many peroxidases in *M. oryzae* is responsive to ROS, including H_2_O_2_.

For the biotrophic pathogens, *Claviceps purpurea* and *Ustilago maydis*, the transcription factor genes *CPTF1* and *YAP1*, respectively, were demonstrated to be required to cope with oxidative stresses^18^,^45^. In *M. oryzae*, an ortholog of *YAP1*, *MoAP1*, was also shown to mediate oxidative stress responses^21^. Loss of these genes universally resulted in attenuated virulence. It is tempting to speculate that these transcription factors, in combination with others, would form a regulatory network shared by peroxidase genes in *M. oryzae*, explaining the common *in planta* transcriptional response among peroxidase genes. In support of this notion, microarray experiments on a *YAP1* deletion mutant showed that at least two peroxidase genes were regulated by *YAP1* in *U. maydis*.

We conducted functional analysis using targeted gene deletion for a set genes selected among the 27 genes. We basically selected genes from clades that did not contain previously characterized genes. Such an approach, in combination with knowledge of previously characterized genes, allowed us to evaluate the roles of peroxidase genes in fungal pathogenesis. Interestingly, all deletion mutants of the seven genes displayed attenuated virulence compared to the wild-type.

We also observed that loss of the selected *M. oryzae* peroxidase genes resulted in decreased peroxidase activity and reduced tolerance to exogenous H_2_O_2_. Our correlation analysis of these attributes (pathogenicity, peroxidase activity, and sensitivity to exogenous H_2_O_2_) suggested that sensitivity to exogenous H_2_O_2_ could be a good proxy for pathogenicity defects (r = 0.76). It is noteworthy that deletion of 13 peroxidase genes to date (6 from previous studies and 7 from this study) led to attenuated virulence, without an exception. Such unanimous results suggest that all peroxidases are required for fungal pathogenesis, although their contributions vary widely.

Among the previously characterized genes, *NOX1* and *NOX2* were involved in regulation at the intracellular ROS level. For the remaining four genes, deletion of individual genes rendered the mutants sensitive to exogenous H_2_O_2._ This sensitivity to H_2_O_2_ correlated with the degree of fungal virulence, with the exception of *CPXB*, deletion of which had no effect on virulence. The reason that these mutants were impaired in virulence was not the loss of ability to detoxify or suppress host-driven ROS, except for *HYR1*. Nevertheless, fortifying the cell wall, as shown in the study of *CATB*, can be, though indirect, a means of coping with the fatal ROS-rampant environment. In most of the mutants in our work developmental changes, including conidiation, conidial germination, and appressorium formation, were not significantly impaired. It is likely that all of the mutants were defective in the penetration or post-penetration phase. We confirmed that, at least in Δ*Moprx1*, the penetration process was not compromised. Considering the reduction in peroxidase activity and H_2_O_2_ tolerance, this suggests that the mutants were less pathogenic due to defects in coping directly or indirectly with host-driven ROS.

During our study, one of the mutants, Δ*Moprx1*, drew our attention because it was almost non-pathogenic to rice plants. *MoPRX1* was, based on sequence similarity, predicted as a gene encoding peroxiredoxin. In mammals, peroxiredoxins are known to be versatile. They possess antioxidant and chaperone-like activities, and, therefore, protect cells from oxidative insults^46^. Moreover, they might directly interact with transcriptional factors such as c-Myc and NF-κB in the nucleus, and be secreted by some cells^30^,^47^. Such versatility of mammalian counterparts and the dramatic defect in *ΔMoprx1* pathogenicity encouraged us to examine this mutant in more detail.

MoPRX1 was homologous to PRX1 (YBL064C) in *S. cerevisiae* (Supplementary Table S1and S3). PRX1 exerts protective antioxidant roles in a cell through its peroxidase activity in detoxifying ROS^48^,^49^,^50^. In *S. cerevisiae*, *PRX1* expression is induced in response to oxidative stress and activation of respiratory pathways^51^. Similarly, the *PRX1* gene in the human pathogenic fungi, *A. fumigatus* and *C. albicans*, was also induced after H_2_O_2_ exposure^52^,^53^. Unlike *S. cerevisiae* and human pathogenic fungi, however, we found that *MoPRX1* expression was not induced in the presence of exogenous H_2_O_2_, but during appressorial development and infection at 78 hpi (Fig. 2). Furthermore, MoPRX1 seemed to be present in cytoplasm regardless of developmental changes. *In silico* analysis of *S. cerevisae* PRX1 and MoPRX1 using PSortII^54^, TargetP^55^, and Mitoprot^56^ predicted unanimously that PRX1 would be localized to mitochondria while MoPRX1 to cytoplasm and mitochondrial-targeting signal (MTS)^57^ was not found in MoPRX1 , suggesting that cytoplasmic localization of MoPRX1 is less likely to be artifact.

This was in contrast to *S. cerevisiae* PRX1, which was shown to localize to mitochondria and *C. albicans* PRX1 that translocated from cytoplasm to the nucleus during hyphal transition. Despite such differences, *MoPRX1* could complement hypersensitivity of the *PRX1* deletion mutant of *S. cerevisiae*, indicating functional conservation as a peroxiredoxin. Although expression of *MoPRX1* was not induced in response to exogenous H_2_O_2_, the deletion mutant was extremely sensitive to this exogenous H_2_O_2_, suggesting that *MoPRX1* might be constitutively expressed to function at the front line against oxidative stresses. Another interesting speculation regarding MoPRX1 is that it mainly functions as a general protective barrier against ROS in cytoplasm, while its orthologs detoxify ROS in tight association with metabolic or developmental cell changes. Our data suggest that the functions of PRX1 as a peroxiredoxin have been co-opted depending on the lifestyle of the fungal species.

Using DAB and H_2_DCFDA staining’s of rice sheaths during infection; we showed that *MoPRX1* is required to incapacitate host-derived ROS *in planta*. Our localization analysis of MoPRX1 raised the question of how MoPRX1 removes host-derived ROS while they are present in the fungal cytoplasm. This is reminiscent of recent reports from studies focusing on *DES1* and *HYR1*^22^,^24^. DES1 and HYR1 were not secreted into the extracellular milieu, yet they could detoxify/suppress ROS generated by the host plant as a defense response. One possible explanation is that *MoPRX1* regulated other ROS-related genes, such as peroxidases and laccases, as suggested in the case of *DES1* and *HYR1*. In support of this, we found that deletion of *MoPRX1* resulted in loss of extracellular peroxidase and laccase activity (Supplementary Fig. S5), as well as causes perturbations in the transcription of other peroxidase genes.

In humans, a peroxiredoxin was shown to function as a molecular chaperone^58^,^59^,^60^. Alternatively, it is, therefore, possible that MoPRX1 also acts as a molecular chaperone that is involved in processing secreted proteins. The second possibility is not mutually exclusive to the first and should be an interesting topic for future studies.

In conclusion, we systematically identified and characterized peroxidase genes in the rice blast fungus. Combining a phylogenetic approach with expression profiling and gene deletion, our work provided not only a comprehensive view of the contributions made by peroxidase genes to fungal pathogenesis but also insights into infection strategy built on evolutionarily conserved peroxidases in the fungus. Furthermore, our study posed important questions regarding how the peroxidase-mediated system is regulated at the transcriptional level and the relationships among peroxidases and novel factors, such as *DES1*. These issues should be addressed in future studies to elucidate ROS-scavenging pathways in fungi pathogenic to plants.

## Materials and Methods

### Identification of peroxidase genes

Putative peroxidase-encoding genes were retrieved from the Fungal Peroxidase Database (fPoxDB; http://peroxidase.riceblast.snu.ac.kr/), which is a fungi-oriented peroxidase genomics platform^44^. The collected protein sequences were used to conduct phylogenetic analysis. The protein sequences were aligned with ClustalW in the MEGA6.0 software with default parameters^61^. Phylogenetic trees were constructed using the neighbor-joining method in the MEGA6.0 software. The peroxidase gene protein structure was obtained from the InterPro database (http://www.ebi.ac.kr/interpro).

### Fungal strains and culture conditions

*Magnaporthe oryzae* KJ201 was obtained from the Center for Fungal Genetic Resources (CFGR) at Seoul National University, Seoul, Korea, and used as the wild-type in this study. This strain and all generated transformants were cultured on oatmeal agar medium (50-g oatmeal per liter with 2% agar (w/v)) or V8-Juice agar medium (4% V8 Juice, pH 6.8) at 25 °C under continuous fluorescent light, cultured in complete liquid medium (0.6% yeast extract, 0.6% casamino acid, and 1% glucose) at 25 °C for 3–4 days with agitation (120 rpm) for genomic DNA extraction. For RNA extraction, all materials were prepared as described previously^62^ (Supplementary Table S5). Hygromycin B-resistant and geneticin-resistant transformants generated by fungal transformation were selected on solid TB3 agar medium (0.3% yeast extract, 0.3% casamino acid, 1% glucose, 20% sucrose (w/v), and 0.8% agar) supplemented with 200 ppm hygromycin B and 800 ppm geneticin. The wild-type and transformants were cultured on complete agar medium (CM), minimal agar medium (MM), C starvation, and N starvation medium to observe growth and colony characteristics^63^. Cell wall biogenesis was examined by imposing stress conditions under 200 ppm Congo Red (CR, Aldrich, 860956) supplementation in CM agar medium. Oxidative stress conditions were elicited in CM agar medium, and amended to final concentrations of 2.5, 5, and 10 mM H_2_O_2_.

### Analysis of transcript levels

Quantitative real-time RT-PCR (qRT-PCR) was employed to measure transcript levels. Total RNA samples and first-strand cDNA were prepared as described previously^62^. qRT-PCR followed Park *et al*.^62^ using each primer pair (Supplementary Table S6). All reactions were performed with more than two biological and three experimental replicates. The *β-tubulin* gene was used as the internal control for normalization. All amplification curves were analyzed with a normalized reporter threshold of 0.1 to obtain the threshold cycle (Ct) values. The comparative ΔΔCt method was applied to evaluate relative quantities of each amplified sample product. Fold changes were calculated as 2^−ΔΔCt^ 62,64. We applied a fold-change cutoff of ≥1.5 for upregulation, and ≤0.5 for downregulation.

### Targeted deletion of seven peroxidase genes and Δ*Moprx1* complementation in *M. oryzae*

Based on seven peroxidase gene sequences in the *M. oryzae* genome, the 5´ (1.2–1.5-kb) and 3´ (1.2–1.5-kb) flanking regions were amplified using primer pairs from each gene: _UF and _UR for the 5´ flanking, and _DF and _DR for the 3´ flanking regions (Supplementary Table S6) from KJ201 genomic DNA. The 1.4-kb HPH marker cassette was amplified from pBCATPH^65^ using primers HPH_F and HPH_R. These three amplicons were fused by double-joint PCR^66^, and the resulting mutant constructs were amplified using the nested primer pair (_UNF and _DNR; as shown in Supplementary Table S6). Fungal protoplasts from wild-type KJ201 were directly transformed using the double-joint PCR product following purification using the standard polyethylene glycol method. Putative gene deletion mutants were screened, and the candidate gene deletion mutants were subsequently purified by single conidia isolation. Southern blot analysis was performed to confirm deletion mutants.

*ΔMoprx1* complementation was achieved by amplifying a 3.6-kb fragment containing the *MoPRX1* open reading frame (ORF), and a 1.3-kb of the 5´ and 1.2 kb of the 3´ flanking regions from wild-type genomic DNA using the *MoPRX1*_UF and *MoPRX1*_DR primers. Geneticin resistance fragment was amplified from PII99^22^ vector plasmid with primers HPH_F (2.1 kb) and HPH_R (2.1 kb) (Supplementary Table S6). The purified 3.6-kb complementation construct was co-transformed with the geneticin fragment into *ΔMoprx1* protoplasts. Putative complemented transformants were selected on TB3 plates amended with 800 ppm geneticin. After genetic purification by single conidium isolation, complements were confirmed by *MoPRX1* gene expression through RT-PCR (Supplementary Fig. S2).

### Nucleic acids manipulation and Southern blotting

Most molecular biology-related techniques, including clone preparation, plasmid DNA, restriction enzyme digestion, and Southern blot analysis, were performed as described previously^67^. Genomic DNA and total RNA were extracted following Park *et al*.^67^ and Park *et al*.^62^, respectively. For PCR screening of generated transformants, genomic DNA was extracted using a rapid and safe DNA extraction method^68^.

### *In vitro* growth assays, monitoring of infectious growth, and pathogenicity assays

Vegetative growth was measured on CM and MM agar plates at 7 and 12 days post incubation (dpi) with three replicates. Melanization, conidiation, conidial size, conidial germination, and infection assays on rice sheath cells, onion epidermis, and rice seedlings were conducted as described previously^69^,^70^. For pathogenicity test,10 ml conidial suspension (5 × 10^4^ spores/ml) containing Tween 20 (250 ppm) was used to spray onto susceptible 3-week-old rice seedlings (*Oryza sativa* cv.Nakdongbyeo). Disease severity was measured at 7 dpi and disease leaf area (DLA) was measured for more accurate evaluation. The disease area and healthy leaf area were measured using the Image J software (http://imagej.nih.gov/ij/) from equal areas of infected leaves of each strain. All experiments were replicated a minimum of three times. For infiltration, a wound inoculation experiment was performed by cutting leaves from plants, which were subsequently wounded with a needle tip prior to inoculation with 40-μl conidial suspension; the same concentration used in spray inoculation. Leaves were placed in a moist box and incubated in a growth chamber. Photographs were taken at 5 dpi. For rice root inoculation, mycelial blocks were plugged on the root surface of seedlings placed on water agar and incubated in a sealed growth chamber. Lesions were observed and photographs were taken at 5 dpi.

### Staining of H_2_O_2_ accumulation in host cells

DAB (3,3´ diaminobenzidine, Sigma, D-8001) staining was conducted as described previously^22^. Excised sheath samples were incubated in 1 mg/ml DAB solution at room temperature for 8 h, and destained with clearing solution (ethanol: acetic acid = 94:4, v/v) for 1 h. H_2_DCFDA staining was also performed with excised rice sheaths following Huang *et al*.^24^. Inoculated excised rice sheaths were incubated for 1 h in 5–20 mM H_2_DCFDA dissolved in DMSO at room temperature, washed with 0.1 mM KCl, 0.1 mM CaCl_2_ (pH 6.0), and maintained at room temperature for 1 h before observation. Fluorescence and DIC micrographs were generated using a Zesis Axio Imager AI fluorescence microscope (Carl Zeiss, Oberkochen, Germany). UV light and eGFP filter were used to detect phenolic compounds and ROS signals after staining with H_2_DCFDA.

### Yeast strain and complementation assays

The *S. cerevisiae* strains YBL064c (*ΔPrx1*) and BY4742 (wild-type) were obtained from EUROSCARF (http://web.uni-frankfurt.de/fb15/mikro/euroscarf/), and maintained on YPD medium. The *MoPRX1* ORF was amplified from first-strand cDNAs of the wild-type with primers *MoPRX1*_ORF_F_HindIII and *Moprx1*_ORF_R_XbaI (Supplementary Table S6), and cloned into the *Hind*III and *Xba*I sites of pYES2 (Invitrogen, Carlsbad, CA, USA). The isolated plasmid pSY259 was transformed into the yeast strain YBL064c (*ΔPrx1*), using the lithium acetate method^71^. Ura3+ transformants were isolated, and the presence of the *MoPRX1* ORF in the transformants was double confirmed by PCR. For complementation assays, cells were cultured overnight, diluted in phosphate-buffered saline and cells were counted using a hemocytometer to adjust the required concentration. The final concentration was adjusted to 2 × 10^3^ cells/μl, and aliquoted in 100-μl samples for each strain. Samples were heated to 50 °C for different time intervals until lethal heat shock was reached, cooled on ice, and 10-μl samples were plated on YPD agar medium. The plates were incubated at 30 °C for 2 days. Cells were counted and survival rate was measured. Oxidant chemical sensitivity was also tested using 10-μl aliquot patch assays containing 10^3^, 10^4^, and 10^5^ cells/μl from overnight cultures, spotted on YPD agar medium supplemented with 3, 4, and 4.5 mM H_2_O_2,_ respectively.

### Measurement of extracellular peroxidase and laccase activities

Extracellular enzyme activity was measured using 3-day-old CM liquid culture filtrate. The measurement was performed by following Chi *et al*.^22^. Peroxidase and laccase activities were measured by combining 1 ml of reaction mixture (50 mM sodium acetate buffer, pH 5.0 and 20 mM ABTS [Sigma, A1888]) with 200-μl culture filtrate, followed by incubation for 5 min at 25 °C. Absorbance was evaluated at a 420-nm wavelength using a spectrophotometer. Three independent biological experiments, with three replicates per experiment, were performed for each test. A mycelial block was placed on CM medium containing 200 ppm Congo Red agar plate for 9 dpi to measure peroxidase secretion. Laccase activity was also monitored on 0.2 mM ABTS agar plate assays with or without 0.5 mM copper sulfate at 4 dpi.

### Cellular localization of MoPRX1::GFP

The MoPRX1::eGFP fusion construct was generated by double-joint PCR. A 2.3-kb genomic fragment, including the putative promoter and full *MoPRX1* ORF region, was amplified with primers *MoPRX1*_UF and *MoPRX1*_ORF_R_eGFP (Supplementary Table S6). The *eGFP* ORF (0.7 kb) with terminator (0.3 kb) was amplified with primers eGFP_F and NC_Term_R from the SK2707 plasmid as a template. The resulting PCR products were fused by double-joint PCR^66^ using primers *MoPRX1*_5NF and NC_Term_NR. The eGFP fusion construct was introduced into *ΔMoprx1* by co-transformation with pII99^22^ plasmid, which carried the geneticin-resistance gene. MoPRX1::eGFP cellular localization was observed in conidia, appressoria and infectious hypha within rice cells at 48 hpi using a fluorescence microscope (Carl Zeiss Microscope Division, Oberkochen, Germany) with a GFP filter.

## Additional Information

**How to cite this article**: Mir, A. A. *et al*. Systematic characterization of the peroxidase gene family provides new insights into fungal pathogenicity in *Magnaporthe oryzae*. *Sci. Rep*. **5**, 11831; doi: 10.1038/srep11831 (2015).

## Supplementary Material

Supplementary Information
