# Supplementary material for: Systematic characterization of the peroxidase gene family provides new insights into fungal pathogenicity in Magnaporthe oryzae
Source: Sci Rep. 2015 Jul 2;5:11831. doi: 10.1038/srep11831 (PMC4488832; doi:10.1038/srep11831)
Supplement: Supplementary Information [file srep11831-s1.pdf]

Supplementary Information

Systematic characterization of the peroxidase gene family provides new insights into fungal pathogenicity in *Magnaporthe oryzae*

Albely Afifa Mir[1], Sook-Young Park[1, 2], Md. Abu Sadat[1,3], Seongbeom Kim[1], Jaeyoung Choi[1,4], Junhyun Jeon[1,5], and Yong-Hwan Lee[1]

[1]Department of Agricultural Biotechnology, Fungal Bioinformatics Laboratory, Center for Fungal Genetic Resources, and Center for Fungal Pathogenesis, Seoul National University, Seoul 151-921, Korea

[2]Current Address: Korean Lichen Research Institute, Sunchon National University, Sunchon 540-742, Korea

[3]Current address: Division of Life Sciences, College of Life Sciences and Bioengineering, Incheon National University, Incheon 406-772, Korea

[4]Current Address: Department of Forest Sciences, University of Helsinki, Helsinki, Finland

[5]Current Address: School of Biotechnology, Yeungnam University, Gyeongsan, Gyeongbuk 712-749, Korea

Corresponding author: Yong-Hwan Lee*

E-mail: yonglee@snu.ac.kr.

Telephone: +82 2 880 4674

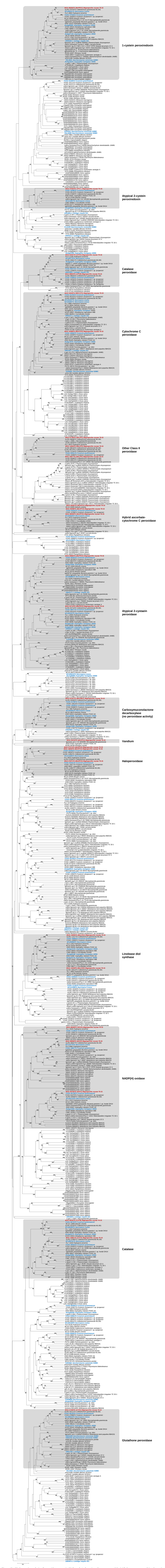

Figure S1. Phylogenetic analysis of peroxidase genes in 29 fungal, 1 chromista, 3 metazoa, and 2 viridiplantae genomes (List shown in supplementary Table S1). A neighbor-joining tree was constructed based on the amino acid sequences of all of the peroxidase genes. The numbers at the nodes indicate bootstrap values (%) in 10,000 bootstrap replicates; only nodes supported by 50 bootstraps or more are shown. Gray boxes are the phylogenetic positions of 27 Magnaporthe oryzae peroxidase genes. Red and blue bold letters denote *M. oryzae* and five selected fungal species, respectively.

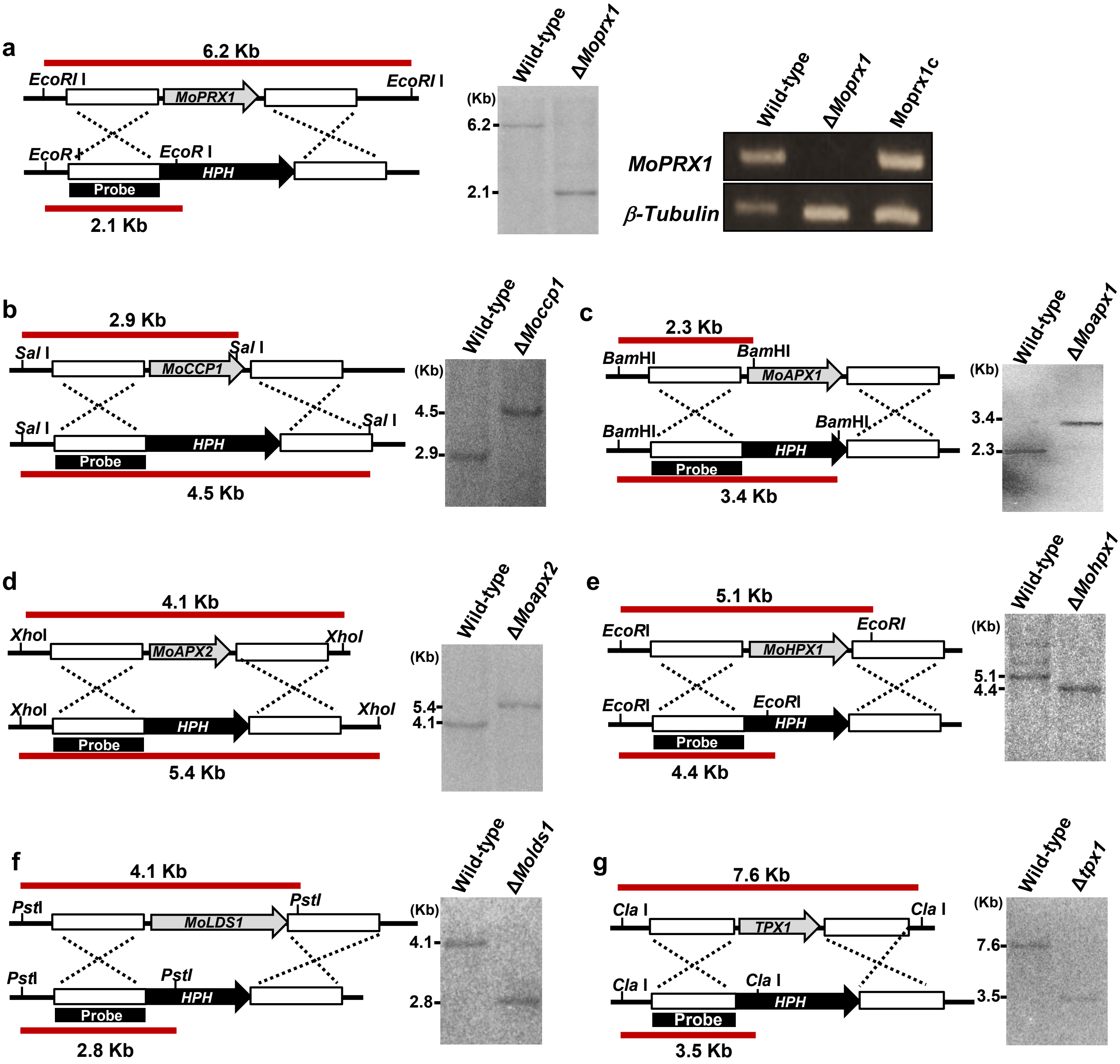

Figure S2. **Generation of deletion mutants for selected *M. oryzae* peroxidase genes.** A total of seven knockout vectors were constructed by PCR amplification. Approximately 1.2~1.5-kb upstream and downstream flanking sequences were amplified using UF/UR and DF/DR, respectively (primer list in Supplementary Table S6), and ligated with the 1.4-kb or 2.1-kb *HPH* cassette. Southern blots analysis of appropriate enzyme-digested genomic DNAs from the wild-type strain and putative gene deletion mutants. The blots were hybridized with each probe shown in Supplementary Fig. S2 (a-g). For the complementation of Δ*Moprx1*, putative complemented transformants were generated and tested for transcriptional expression of *MoPRX1* gene by RT-PCR (a).

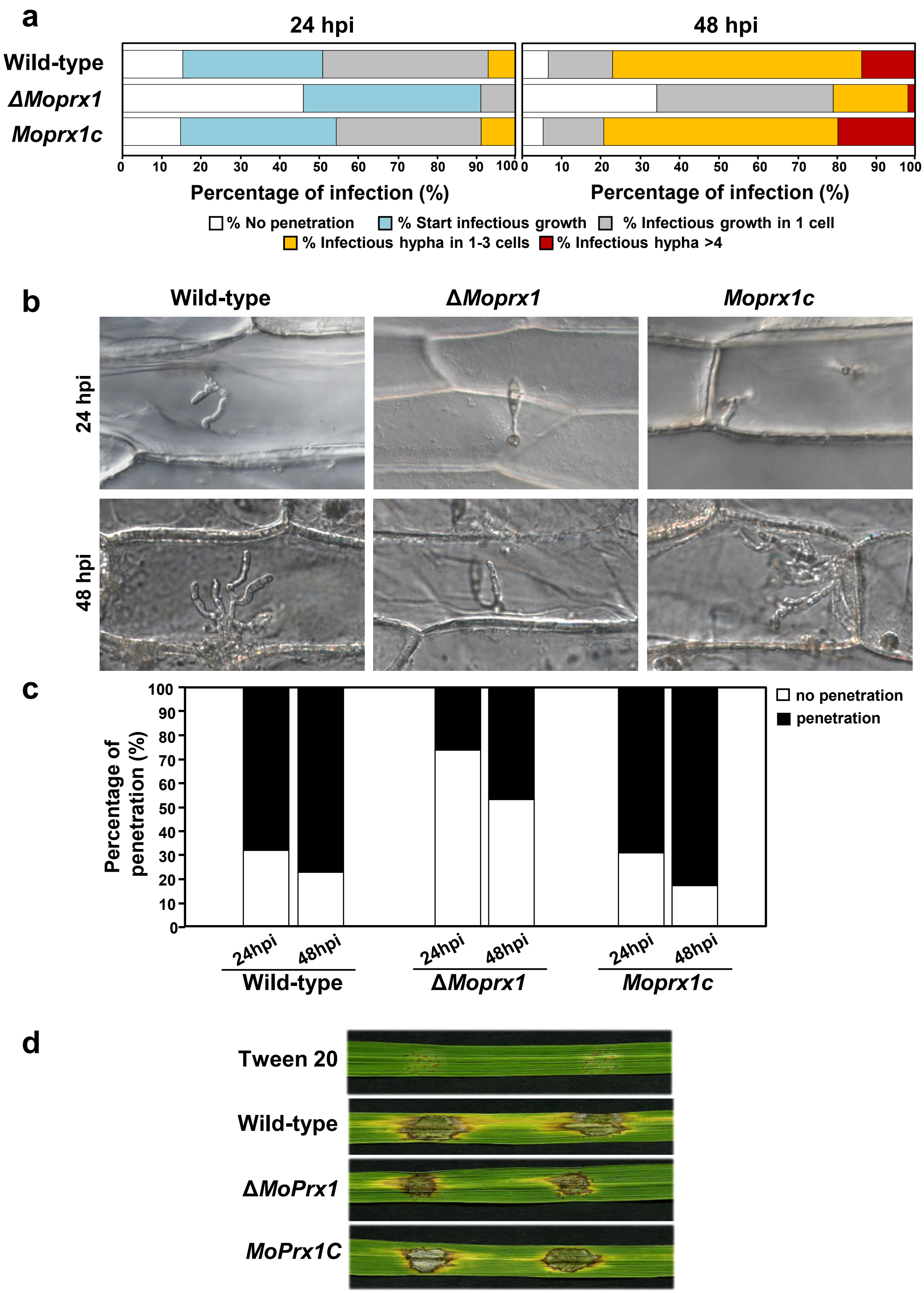

Figure S3. **Penetration percentage and pathogenicity of Δ*Moprx1* on rice sheaths and leaves, and onion epidermal cells.** (a) Quantitative measurement of rice sheath assay by Δ*Moprx1*. Frequency was measured by counting three replicates of 75-100 appressoria. (b) Appressorial penetration assay on onion epidermal cell by drop inoculation with 2×10^4 conidia/ml and observed at 24 and 48 hpi. Images were captured using a 20-ms exposure to transmitted light with a DIC filter. (c) Percentage of appressorial penetration of onion epidermal cells. Data were obtained by counting three replicates of >100 appressoria. (d) Infiltration experiment on rice leaves. The conidial suspension (5×10^4 conidia/ml) was used as an inoculum. Photograph was taken at 5 dpi.

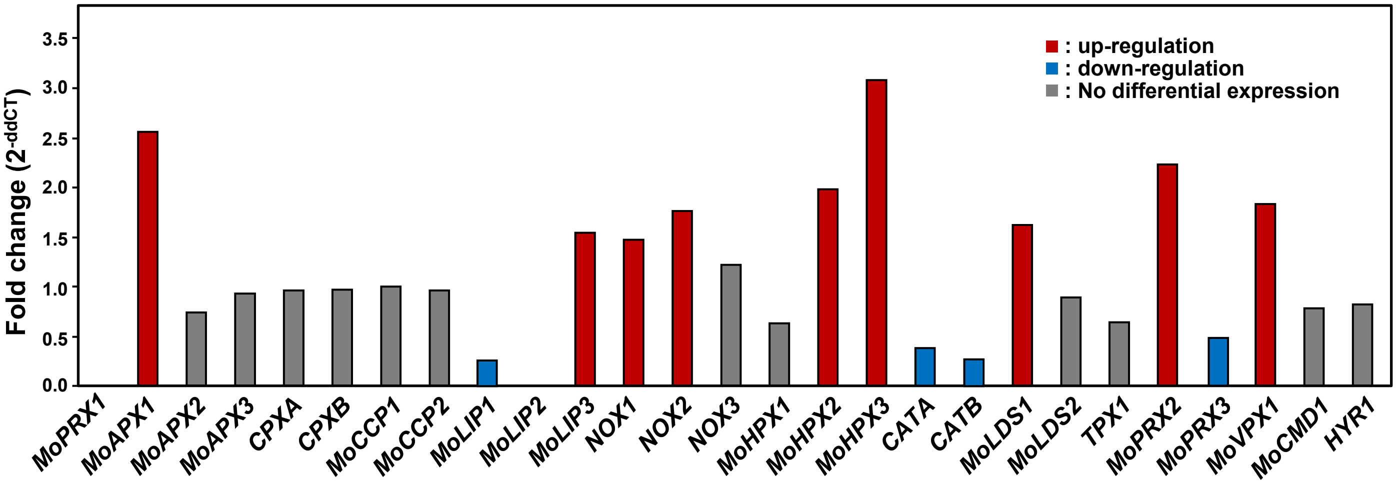

Figure S4. Expression profiles of 27 *M. oryzae* peroxidase genes in Δ*Moprx1*. Upregulated genes (more than 1.5-fold) are indicated by red bars, and downregulated genes (less than 0.5-fold) are denoted by blue bars. The genes not showing differential expression are marked in gray.

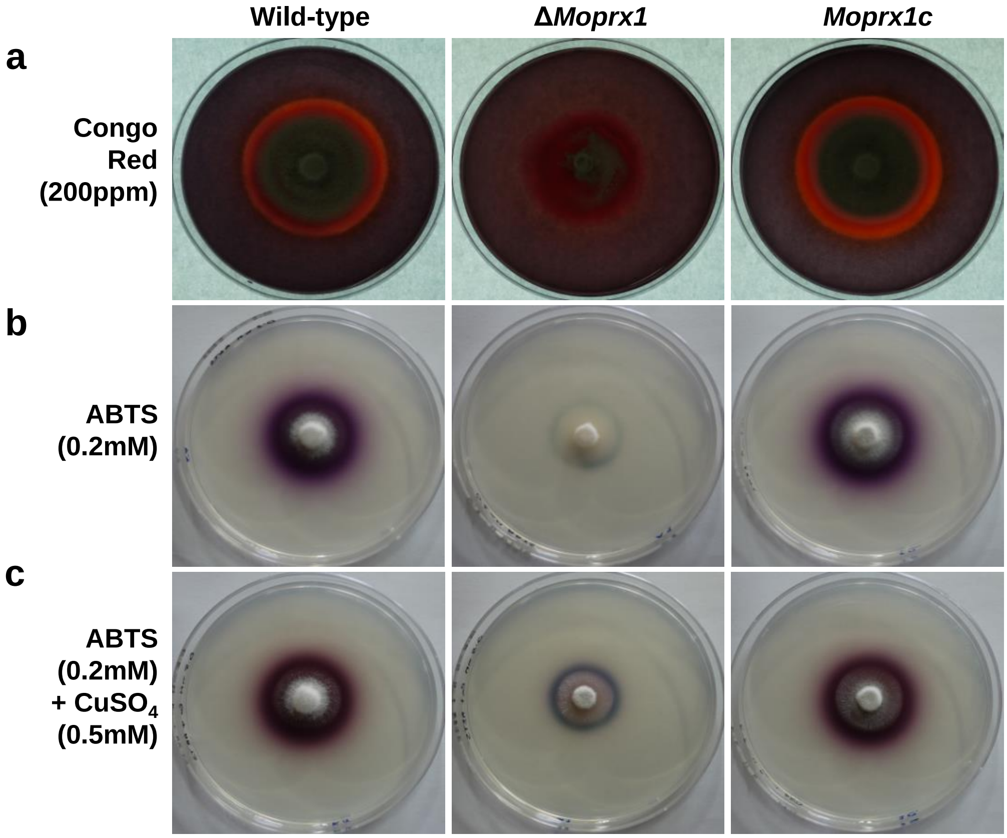

Figure S5. **Extracellular enzyme activity in Δ*Moprx1*.** The wild-type, Δ*Moprx1*, and Moprx1c strains were inoculated on complete media (CM) containing (a) 200 ppm Congo Red, and (b) 0.2 mM ABTS, and (c) 0.2 mM ABTS with 0.5 mM $CuSO_4$. The 5-mm-diameter mycelial blocks were inoculated onto media, and incubated at 25°C. ABTS discoloration was observed at 4 dpi and Congo Red discoloration at 9 dpi.

Table S1. Peroxidase genes from 29 fungal, thee metazoan, two viridiplantae and one chromistal genomes

Kingdom	Phylum/ Subphylum	Species name	No. of Peroxidses	Haem peroxidase						Non-haem peroxidase			
				Animal peroxidase-Cyclooxygenase superfamily	Catalase superfamily	Class I	ClassII	DyP-type peroxidase	NAD(P)H oxidase superfamily	Alkylhydroperoxidase D-like superfamily	Glutathione peroxidase	Haloperoxidase	Peroxiredoxin
Chromista	Oomycota	*Phytophthora infestans*	31	0	2	4	0	7	4	0	7	0	7
Fungi	Ascomycota	*Aspergillus fumigatus* Af293	25	3	4	3	0	4	1	2	1	0	7
		Aspergillus nidulans	25	3	4	3	0	5	1	2	1	0	6
		Blumeria graminis f.sp. *hordei*	14	2	2	3	0	0	2	0	1	0	4
		Botrytis cinerea	30	2	6	4	0	9	1	1	1	0	6
		Candida albicans SC5314	14	0	1	2	0	0	0	0	4	0	7
		*Coccidioides immitis*RS	15	3	2	3	0	0	2	1	1	0	3
		Colletotrichum graminicola M1.001	34	4	4	4	7	5	3	1	1	0	5
		Fusarium graminearum	31	4	5	5	1	5	3	2	1	0	5
		Fusarium oxysporum	45	7	6	8	1	10	3	3	1	0	6
		Histoplasma capsulatum H88	18	3	3	2	0	2	2	1	1	0	4
		Magnaporthe oryzae 70-15	27	2	2	7	3	3	3	1	1	1	4
		Mycosphaerella graminicola	42	4	3	2	2	18	2	2	1	1	7
		Neurospora crassa	20	3	3	4	0	2	2	1	1	0	4
		Podospora anserina	23	2	4	3	1	5	3	1	1	0	3
		Saccharomyces cerevisiae S288C	11	0	2	1	0	0	0	0	3	0	5
		*Schizosaccharomyces pombe*132	5	0	1	0	0	0	0	0	1	0	3
	Basidiomycota	*Cryptococcus neoformans* var. *grubii* H99	14	0	4	2	0	0	0	0	3	0	5
		Heterobasidion irregulare TC 32-1	32	2	3	5	7	6	2	2	0	0	5
		Laccaria bicolor	23	2	1	2	1	7	2	1	1	0	6
		Melampsora larici-populina 98AG31	39	3	6	2	0	18	5	1	1	0	3
		Phanerochaete chrysosporium	39	3	5	1	16	3	2	1	1	0	7
		Puccinia graminis f. sp. *tritici*	24	0	4	2	0	12	2	0	1	0	3
		Serpula lacrymans S7.3	19	2	4	1	0	3	2	0	1	0	6
		Ustilago maydis 521	14	1	0	3	0	3	0	1	1	0	5
	Blastocladiomycota	*Allomyces macrogynus*	21	0	0	4	0	0	7	0	4	0	6
	Chytridiomycota	*Batrachochytrium dendrobatidis* JAM81	11	0	3	2	0	0	1	0	1	0	4
	Microsporidia	*Encephalitozoon cuniculi*	2	0	0	0	0	0	0	0	1	0	1
	Zygomycota	*Phycomyces blakesleeanus*	14	0	2	2	0	0	0	0	2	0	8
		Rhizopus oryzae	19	0	4	3	0	2	0	0	3	0	7
Metazoa	Arthropoda	*Dorosophila melanogaster*	20	0	2	0	0	0	2	0	4	0	12
	Chordata	*Homo sapiens*	53	0	1	0	0	0	19	0	13	0	20
	Nematoda	*Caenorhabditis elegans*	16	0	3	0	0	0	2	0	7	0	4
Viridiplantae	Streptophyta	*Arabidopsis thaliana*	63	3	7	18	0	0	14	0	9	0	12
		Oryza sativa (japonica cultivar-group)	63	4	6	18	0	0	17	0	7	0	11
		**Total**	896	62	109	123	39	129	109	24	88	2	211

Table S2. Predicted peroxidase genes in *M. oryzae*

	Class	Type	Locus	Protein Length	Signal peptide	Annotation
Haem	Class I peroxidase	Hybrid Ascorbate-Cytochrome C peroxidase	MGG_00461	476		hypothetical protein, *MoAPX1*
			MGG_08200	804	V	hypothetical protein, *MoAPX2*
			MGG_09398	548	V	hypothetical protein, *MoAPX3*
		Catalase peroxidase	MGG_04337	750		catalase-peroxidase 1, *CPXA*
			MGG_09834	786	V	catalase-peroxidase 2, *CPXB*
		Cytochrome C peroxidase	MGG_04545	362		cytochrome c peroxidase, *MoCCP1*
			MGG_10368	300		cytochrome c peroxidase, *MoCCP2*
	Class II peroxidase	Other class II peroxidase	MGG_07790	474	V	ligninase H2, *MoLIP1*
			MGG_10877	264	V	hypothetical protein, *MoLIP2*
			MGG_14940	428		ligninase C, *MoLIP3*
	NAD(P)H oxidase superfamily	NADP(H) oxidase	MGG_00750	553		cytochrome b-245 heavychain subunit beta, *NOX1*
			MGG_06559	582		cytochrome b-245 heavychain subunit beta, *NOX2*
			MGG_08299	849		hypothetical protein, *NOX3*
	DyP-type peroxidase	Haloperoxidase (haem)	MGG_16840	242	V	hypothetical protein, *MoHPX1*
			MGG_07574	257	V	hypothetical protein, *MoHPX2*
			MGG_07871	241	V	hypothetical protein, *MoHPX3*
	Catalase superfamily	Catalase	MGG_10061	743		catalase-1, *CATA*
			MGG_06442	715	V	catalase-3, *CATB*
	Animal peroxidase	Linoleate diol synthase (PGHS like)	MGG_10859	1153		heme peroxidase, *MoLDS1*
			MGG_13239	1171		linoleate diol synthase, *MoLDS2*
Non haem	Peroxiredoxin	1-Cysteine peroxiredoxin	MGG_08256	224		mitochondrial peroxiredoxin PRX1, *MoPRX1*
		Atypical 2-Cysteine peroxiredoxin (type Q, BCP)	MGG_07503	314		thioredoxin peroxidase, *TPX1*
		Atypical 2-Cysteine peroxiredoxin (type II &V)	MGG_00860	184		AphC/TSA family protein, *MoPRX2*
			MGG_02710	168		peroxiredoxin type-2, *MoPRX3*
	Haloperoxidase	Vanadium chloroperoxidase	MGG_02210	595		vanadium chloroperoxidase, *MoVPX1*
	Alkyhydroperoxidase D-like superfamily	Carboxymuconolactone decarboxylase (no peroxidase activity)	MGG_01347	243		4-carboxymuconolactone decarboxylase, *MoCMD1*
	Glutathione peroxidase	Fungi-Bacteria glutathione peroxidase	MGG_07460	172		peroxiredoxin HYR1, *HYR1*

Table S3: List of 27 putative peroxidase proteins in *Magnaporthe oryzae* and Blast Matrix results compared to those from 35 organisms

sub-family	Protein name	Pi *	Af1	An	Bg	Bc	Cas	Ci	Cg	Fg	Fol	Hc8	Mo	Mgr	Nc	Pa	Sc	Sp	Cn	Ha	Lb	Mlp	Pc	Pg	Sl7	Um5	Am	BdJ	Ecu	Pbl	Ro	Dm	Hs	Ce	Ath	Osj
	Kingdom	Chromista	Fungi																													Metazoa			Viridiplantae	
	Phylum	Oomycota	Ascomycota																Basidiomycota								Blastocladiomycota	Chytridiomycota	Microsporidia	Zygomycota		Arthropoda	Chordata	Nematoda	Streptophyta	
	Species	Pi *	Af1	An	Bg	Bc	Cas	Ci	Cg	Fg	Fol	Hc8	Mo	Mgr	Nc	Pa	Sc	Sp	Cn	Ha	Lb	Mlp	Pc	Pg	Sl7	Um5	Am	BdJ	Ecu	Pbl	Ro	Dm	Hs	Ce	Ath	Osj
Hybrid Ascorbate-Cytochrome C peroxidase	MoAPX1	-	-	-	-	±	-	±	±	±	±	-	±	-	±	±	-	-	-	±	±	-	-	-	-	-	-	-	-	-	-	-	-	-	-	-
	MoAPX2	-	±	±	±	±	±	±	±	±	±	±	±	±	±	±	±	-	±	±	±	±	±	±	±	±	±	±	-	±	±	±	±	±	±	±
	MoAPX3	±	±	±	±	±	±	±	±	±	±	±	±	±	±	±	±	-	±	±	±	±	±	±	±	±	±	±	-	±	±	-	-	-	±	±
Catalase peroxidase	CPXA	±	±	±	±	±	±	±	±	±	±	±	±	±	±	±	±	-	±	±	±	±	±	±	±	±	±	±	-	±	±	-	-	-	±	±
	CPXB	±	±	±	±	±	±	±	±	±	±	±	±	±	±	±	±	-	±	±	±	±	±	±	±	±	±	±	-	±	±	-	-	-	±	±
Cytochrome C peroxidase	MoCCP1	±	±	±	±	±	±	±	±	±	±	±	±	±	±	±	±	-	±	±	±	±	±	±	±	±	±	±	-	±	±	-	-	-	±	±
	MoCCP2	±	±	±	±	±	±	±	±	±	±	±	±	±	±	±	±	-	±	±	±	±	±	±	±	±	±	±	-	±	±	-	-	-	±	±
Other class II peroxidase	MoLIP1	-	-	-	-	-	-	-	±	±	±	-	±	±	-	±	-	-	-	±	±	-	±	±	-	-	±	±	-	-	-	-	-	-	±	±
	MoLIP2	±	±	-	±	±	-	-	±	±	±	±	±	±	-	±	-	-	-	±	±	-	±	-	±	-	±	±	-	-	-	-	-	-	±	±
	MoLIP3	-	-	-	-	-	±	-	±	±	±	-	±	±	-	±	-	-	-	±	±	-	±	-	-	-	-	-	-	-	-	-	-	-	±	±
NADP(H) oxidase	NOX1	±	±	±	±	±	±	±	±	±	±	±	±	±	±	±	-	±	±	±	±	±	±	±	±	-	±	±	-	-	±	±	±	±	±	±
	NOX2	±	±	±	±	±	±	±	±	±	±	±	±	±	±	±	±	±	±	±	±	±	±	±	±	±	±	±	-	-	±	±	±	±	±	±
	NOX3	±	±	±	±	±	±	±	±	±	±	±	±	±	±	±	±	±	±	±	±	±	±	±	±	-	±	±	-	-	±	±	±	±	±	±
Haloperoxidase (haem)	MoHPX1	±	±	±	-	±	-	-	±	±	±	±	±	±	±	±	-	-	-	±	±	±	±	±	±	±	-	-	-	-	±	-	-	-	-	-
	MoHPX2	±	±	±	-	±	-	-	±	±	±	±	±	±	±	±	-	-	-	±	±	±	±	±	±	±	-	-	-	-	±	-	-	-	-	-
	MoHPX3	±	±	±	-	±	-	-	±	±	±	±	±	±	±	±	-	-	-	±	±	±	±	-	±	±	-	-	-	-	±	-	-	-	-	-
Catalase	CATA	±	±	±	±	±	±	±	±	±	±	±	±	±	±	±	±	±	±	±	±	±	±	±	±	-	-	±	-	±	±	±	±	±	±	±
	CATB	±	±	±	±	±	±	±	±	±	±	±	±	±	±	±	±	±	±	±	±	±	±	±	±	-	-	±	-	±	±	±	±	±	±	±
Linoleate diol synthase (PGHS like)	MoLDS1	-	±	±	±	±	-	±	±	±	±	±	±	±	±	±	-	-	-	±	±	±	±	-	±	±	±	-	-	-	±	-	±	-	-	±
	MoLDS2	-	±	±	±	±	-	±	±	±	±	±	±	±	±	±	-	-	-	±	±	±	±	-	±	±	-	-	-	-	±	-	±	-	-	-
Peroxiredoxin	MoPRX1	±	±	±	±	±	±	±	±	±	±	±	±	±	±	±	±	±	±	±	±	±	±	±	±	±	±	±	±	±	±	±	±	±	±	±
	TPX1	±	±	±	±	±	±	±	±	±	±	±	±	±	±	±	±	±	±	±	±	±	±	±	±	±	±	±	±	±	±	±	±	±	±	±
	MoPRX2	-	±	±	±	±	±	±	±	±	±	±	±	±	±	±	±	±	±	±	±	±	±	±	±	±	-	±	-	±	±	±	±	-	±	±
	MoPRX3	-	±	±	±	±	±	±	±	±	±	±	±	±	±	±	±	±	±	±	±	±	±	±	±	±	-	±	-	±	±	±	±	-	±	±
Vanadium chloroperoxidase	MoVPX1	-	-	-	-	-	-	-	-	-	-	-	±	±	-	±	-	-	-	-	-	-	-	-	-	-	-	-	-	-	-	-	-	-	-	-
Carboxymuconolactone decarboxylase	MoCMD1	-	±	±	-	±	-	±	±	±	±	±	±	±	±	±	-	-	-	±	±	±	±	±	-	±	-	-	-	-	-	-	-	-	-	-
Fungi-Bacteria glutathione peroxidase	HYR1	±	±	±	±	±	±	±	±	±	±	±	±	±	±	±	±	±	±	-	±	±	±	±	±	±	±	±	±	±	±	±	±	±	±	±

± : 1e-100 ; ± : 1e-50 ; ± : 1e-30 ; ± : 1e-10 ; - : None

*Abbreviation for fungal species and other organisms: Pi: *Phytophthora infestans*; Af1: *Aspergillus fumigatus* Af293; An: *Aspergillus nidulans*; Bg: *Blumeria graminis* f.sp. hordei; Bc: *Botrytis cinerea*; Cas: *Candida albicans* SC5314; Ci: *Coccidioides immitis*; Cg: *Colletotrichum graminicola* M1.001; Fg: *Fusarium graminearum*; Fol: *Fusarium oxysporum*; Hc8: *Histoplasma capsulatum* H88; Mo: *Magnaporthe oryzae* 70-15; Mgr: *Mycosphaerella graminicola*; Nc: *Neurospora crassa*; Pa: *Podospora anserina*; Sc: *Saccharomyces cerevisiae* S288C; Sp: *Schizosaccharomyces pombe*; Cn: *Cryptococcus neoformans* variety gatti (serotype A) ; Ha: *Heterobasidion annosum*; Lb: *Laccaria bicolor*; Mlp: *Melampsora larici-populina* 98AG31; Pc: *Phanerochaete chrysosporium*; Pg: *Puccinia graminis*; Sl7: *Serpula lacrymans* S7.3; Um5: *Ustilago maydis* 521; Am: *Allomyces macrogynus*; Bdj: *Batrachochytrium dendrobatidis* JAM81; Ec: *Encephalitozoon cuniculi*; Pbl: *Phycomyces blakesleeanus*; Ro: *Rhizopus oryzae*; Dm: *Dorosophila melanogaster*; Hs: *Homo sapiens*; Ce: *Caenorhabditis elegans*; Ath: *Arabidopsis thaliana*; Osj: *Oryza sativa* japonica.

Table S4. Characterization of transformants including deletion mutants for seven peroxidase genes in *M. oryzae* pathogenicity-related phenotypes

Strain	Conidiation (10^5 / ml) [a]	Conidial germination (%) [b]	Appressorium formation (%) [c]
Wild-type	55.7±1.5^{E} [d]	97.7±1.5^{B}	97.3±1.5^{BC}
Δ*Moprx1*	42.7±7.5^{B}	96.7±1.2^{AB}	93.0±3.6^{A}
Moprx1c	56.3±1.5^{E}	94.3±1.5^{A}	96.0±1.0^{ABC}
Δ*Moccp1*	49.0±1.0^{CD}	96.3±0.6^{AB}	95.0±2.6^{ABC}
Δ*Moapx1*	57.3±1.2^{E}	95.7±0.6^{AB}	94.7±2.1^{AB}
Δ*Moapx2*	44.7±2.3^{BC}	94.7±1.5^{A}	97.7±0.6^{BC}
Δ*Mohpx1*	50.3±1.5^{D}	97.7±0.6^{B}	98.3±0.6^{C}
Δ*Molds1*	47.0±1.0^{BCD}	95.7±1.5^{AB}	95.0±1.0^{ABC}
Δ*tpx1*	10.7±2.1^{A}	96.7±1.5^{AB}	96.0±1.0^{ABC}

[a] Conidiation was measured by counting the number of conidia collected with 5 ml of sterilized distilled water from 7-day-old V8 juice agar plate; [b]Percentage of conidial germination on hydrophobic surfaces was measured under a light microscope with three replicates; [c] Percentage of appressorium formation on hydrophobic surfaces was measured using germinated conidia with three replicates; [d] Duncan's test was used to determine significance at the 95% probability level. The same letters in a column showed no significant difference.

Table S5. Total RNA was extracted from five conditions including infection-related developments (conidia, germinated conidia, appressoria, and infectious growth) and oxidative stress treatment

Categories	Conditions	Conc.	Treated time	Basal medium for treatment
Infection-related developments	Conidia<br>Germinated conidia<br>Appressoria<br>78 hpi on rice			
Oxidative stress	H_2O_2	2.5 mM	4 h	CM

Note: Germinated conidia and appressoria were harvested after 4 h and 16 h, respectively, on hydrophobic surface for RNA extraction.

Table S6. List of primers used in this study

Primer name	Sequence (5´ → 3´)
**Knock-out constructs**	
MoPRX1	
*MoPRX1*_UF	TTC CAG GGG TAC TAT GGC AAC AA
*MoPRX1*_UR	CCT CCA CTA GCT CCA GCC AAG CC AAT CCG GGC CCA GTT TAG GTT TAT
*MoPRX1*_DF	GTT GGT GTC GAT GTC AGC TCC GGA G TCA ATA TGG CGC CGC AGC AGT AAT
*MoPRX1*_DR	GTACTCGCCTGCCCTGCCTATCTT
*MoPRX1*_UNF	GCTGCTAAAGGGATTGGCTACTCA
*MoPRX1*_DNR	ATGGTCGCCGCTATTTGCTTATG
*MoPRX1*_UF_F	ATAAACCTAAACTGGGCCCGGATT
*MoPRX1*_DF_R	ATTACTGCTGCGGCGCCATATTGA
MoCCP1	
*MoCCP1*_UF	TCT CGT TCC TGC AGA TGC CGT C
*MoCCP1*_UR	CCT CCA CTA GCT CCA GCC AAG CC TGA GGT CTC TGA CTT CAG TGG TAG GT
*MoCCP1*_DF	GTT GGT GTC GAT GTC AGC TCC GGA G TTG GGG AGG ACT TGA TGG TTA GAC TTG
*MoCCP1*_DR	CAG CCT GGA CTC GAG CAA CAC
*MoCCP1*_UNF	GCC GGA ACT TCC AGT TTG AAG ACA ACT ACC TA
*MoCCP1*_DNR	AAA CTG AGC GAG GAC TAC GAC GCC
*MoCCP1*_UR_F	ACC TAC CAC TGA AGT CAG AGA CCT CA
*MoCCP1*_DF_R	CAA GTC TAA CCA TCA AGT CCT CCC CAA
MoAPX1	
*MoAPX1*_UF	CAG TGG AGG AAT CAG CAA CTC TCA GC
*MoAPX1*_UR	CCT CCA CTA GCT CCA GCC AAG CC CCA TTG CCT TTA TGC GGA AGT TCT TGT
*MoAPX1*_DF	GTT GGT GTC GAT GTC AGC TCC GGA G CAT GAG CGC GTT TCA CAG GAC TC
*MoAPX1*_DR	TGA CAC GCC AAG ACC TAC CAC AGA G
*MoAPX1*_UNF	AAG GGG AAA CAT TGC GCA GAC ATC ACA
*MoAPX1*_DNR	CTT GCC AGC CTA TCT TGT CCA GTA GTG G
*MoAPX1*_UR_F	ACA AGA ACT TCC GCA TAA AGG CAA TGG
*MoAPX1*_DF_R	GAG TCC TGT GAA ACG CGC TCA TG
MoAPX2	
*MoAPX2*_UF	GCG GCT ATA GAA AGA TCC GGC ATG AAT
*MoAPX2*_UR	CCT CCA CTA GCT CCA GCC AAG CC ACA GCA GAT TGT GTG GTG ATG CAC
*MoAPX2*_DF	GTT GGT GTC GAT GTC AGC TCC GGA G GGA TGA GAA TGA GCC AAG CAG GGA ATT
*MoAPX2*_DR	TCG GAG ACG CCT AGC CTT CCC TA
*MoAPX2*_UNF	AAG GTC TCT TAA GGC ACA TTT CGT GCG G
*MoAPX2*_DNR	TGC CCC TAG AAC TCG TTA GTC AGT CTA GTT CG
*MoAPX2*_UR_F	GTG CAT CAC CAC ACA ATC TGC TGT
*MoAPX2*_DF_R	AAT TCC CTG CTT GGC TCA TTC TCA TCC
MoHPX1	
*MoHPX1*_UF	TCGTGAGAGGTGAGGACAATAAAC
*MoHPX1*_UR	CCT CCA CTA GCT CCA GCC AAG CC CTG CCA AAG GAC GAA GGA TTG AAA
*MoHPX1*_DF	GTT GGT GTC GAT GTC AGC TCC GGA G ACT GCT GTT TGA GAT GTC CA
*MoHPX1*_DR	CAA CAC GCA TGA CTC CAC CTA CAA
*MoHPX1*_UNF	TGG CTA GAC ATC TGA GGT TAT
*MoHPX1*_DNR	CTG GTT CGA GGA TGT TTA GTT
*MoHPX1*_UR_F	TTT CAA TCC TTC GTC CTT TGG CAG
*MoHPX1*_DF_R	TGG ACA TCT CAA ACA GCA GT
MoLDS1	
*MoLDS1*_UF	GGT AAG TAA CAT GCT AGG CAT TTG GAG TCT CG
*MoLDS1*_UR	CCT CCA CTA GCT CCA GCC AAG CC GCA ATG GGA ACG CCA TCG TGT ATT
*MoLDS1*_DF	GTT GGT GTC GAT GTC AGC TCC GGA G TGA CTG TTG GTG GGA TAA GAC TGA ATG AA

*MoLDS1*_DR	GAC TGG AAA GTG CCA AGT ATC CTG CTA
*MoLDS1*_UNF	GCG GTC TGA TAG ACC AAT TAA TAG CGA GGC TAA C
*MoLDS1*_DNR	ACA TTC AGG GAC AAT GAA TAC GTC CCT GCA G
TPX1	
*TPX1*_UF	GCG GTT GAA TCT CGG ACA TA
*TPX1*_UR	CCT CCA CTA GCT CCA GCC AAG CC AGG GTA TGG CTC ACG AAA CTA AG
*TPX1*_DF	GTT GGT GTC GAT GTC AGC TCC GGA G TGT AGG ATG AGC GAC GAT AGT
*TPX1*_DR	AAG TTC GTC GAG GTT TCG GA
*TPX1*_UNF	CGA ATT GAA GCA CGC TGA GAG GTT
*TPX1*_DNR	TTG ACC TGT TCT CGC GCT TCT TTG
*TPX1*_UR_F	CTT AGT TTC GTG AGC CAT ACC CT
*TPX1*_DF_R	ACT ATC GTC GCT CAT CCT ACA
**Hygromycin phosphotransferase**	
*HPH*_F (1.4 Kb)	GGCTTGGCTGGAGCTAGTGGAGG
*HPH*_R (1.4Kb)	GTTGGTGTCGATGTCAGCTCCGGAG
*HPH*_F (2.1 Kb)	AGAAGATGATATTGAAGG
*HPH*_R (2.1Kb)	GCACAGGTACACTTGTTTAGAG
SYP_HPH_R	ATGCCTGAACTCACGCGACGTCTGTCG
**GFP tagging**	
*MoPRX1*_ORF_R_eGFP	TCGCCCTTGCTCACCATCTCCT TGGGCAG
eGFP_F	ATGGTGAGCAAGGGCGAGGAG
NcTerm_R	ATCATCATGCAACATGCA
NcTerm_R_nested	GATGTATTAAGAGTATAGGGGTC
*MoPRX1*_ORF_F_HindIII	AAGCTTATGGCCGAAGAGCAGCGT
*MoPRX1*_ORF_R_XbaI	TCTAGACTACTCCTTGGGCAGCGGCGTA
**qRT-PCR**	
β-Tubulin	
TubF	CTCCAGGGTTTCCAGATCAC
TubR	CCTCACCAGTGTACCAATGC
*MoAPX1*_qRT_F	CTTTCAGCCATGGTGTTTCTG
*MoAPX1*_qRT_R	ATGTCTTCCAGCTCATCGTG
*MoAPX2*_qRT_F	CAGGACTTGAAGACACTTGGG
*MoAPX2*_qRT_R	GTAACAGTGAAGGACGAGATACC
*MoAPX3*_qRT_F	ACGCCAGCATCTTTTACGAG
*MoAPX3*_qRT_R	CAAGGTCTCCAAACGTCGAG
*CPXA*_qRT_F	TGTTCCCGTTCCATTCACTC
*CPXA*_qRT_R	GATCTGTTCCGTGGTAACTCG
*CPXB*_qRT_F	TGACCTGATTGACGATGCC
*CPXB*_qRT_R	CTTGTCAGAGTTGCGGTAGG
*MoCCP1*_qRT_F	GATGGTCCTAAGCAGTACGTC
*MoCCP1*_qRT_R	CGGCAGAGAAGTCCTTGAAG
*MoCCP2*_qRT_F	ACGGGTCTGAAGCAGTTTG
*MoCCP2*_qRT_R	GAAATCCGCAAAGAACAGGTC
*MoLIP1*_qRT_F	GCGTCCCACACTTCTCAC
*MoLIP1*_qRT_R	GCTTGTCGTGGAAGTTTTCG
*MoLIP2*_qRT_F	CGTCAATGGTAACAAACTCTGC
*MoLIP2*_qRT_R	AGGAAAGCAGTCATGGAAGG

*MoLIP3*_qRT_F	CTTCACCTCTTGACTCAACCG
*MoLIP3*_qRT_R	ATGGAGGGATAAATGTCGGTG
*NOX3*_qRT_F	ATCAACACCCAGAGAAACCC
*NOX3*_qRT_R	GTACACTACATTCGGGTCATCG
*NOX2*_qRT_F	GAGAGGCAGAAGGAACTTACG
*NOX2*_qRT_R	TCCTTGACAGCATAGTTGACG
*NOX1*_qRT_F	ATTGATGGTCCCTATGGTGC
*NOX1*_qRT_R	ACCAATACCAGTTCCAATGAGG
*MoHPX1*_qRT_F	GGAAGGACTGGGTCAAGATTC
*MoHPX1*_qRT_R	TTTCCTGGGCTGTGATCTTG
*MoHPX2*_qRT_F	TTCACCTTTATCGAGTCGGC
*MoHPX2*_qRT_R	TGCTCGAAAGGAAGACGTTC
*MoHPX3*_qRT_F	CGGGAGTGGTTGAAGGTTATT
*MoHPX3*_qRT_R	CCTGCTCAAACTTGTGGAAAC
*CATA*_qRT_F	ACTTTAGCAACATCACCTCGG
*CATA*_qRT_R	GTCGGTATAGAATCTCGTGGC
*CATB*_qRT_F	CTTTCCTCTCACTTCCTCTTCC
*CATB*_qRT_R	AGTCAACTAACCCTTGGTGTG
*MoLDS1*_qRT_F	CTACAACCATCCCTTCAACCC
*MoLDS1*_qRT_R	GAGTCAAAGATCAGCCCAGG
*MoLDS2*_qRT_F	CTTTCCCTAACCACTACGCC
*MoLDS2*_qRT_R	GACCCAGACTCTCCATAATCTTC
*MoPRX1*_qRT_F	TGGATCAAGGACATCAACGAC
*MoPRX1*_qRT_R	GCGGATGGTAAAGGCAATTC
*TPX1*_qRT_F	CCCGTGGAGTGTTCGTTATTG
*TPX2*_qRT_R	CCTTAGCCTCTTCAACCTTGG
*MoPRX2*_qRT_F	AGCCACATCCCTTCATACATG
*MoPRX2*_qRT_R	GCCCAAGCCTTCATAACAAAG
*MoPRX3*_qRT_F	TTGTCATCGTCAGTGTTCCG
*MoPRX3*_qRT_R	CTTCTCGACGTAGCTCTTGAG
*MoVPX1*_qRT_F	TCACTCCATACCTCCCTGAG
*MoVPX1*_qRT_R	GTTTTCCTGGCGATGCTTG
*MoCMD1*_qRT_F	CTCTCGGATAAGCAGTGGG
*MoCMD1*_qRT_R	GTGATCTCGACAACCTCACG
*HYR1*_qRT_F	GACTACAAGGGCAAGGTCG
*HYR1*_qRT_R	GAAGCCGAGGATGGTAAAGTC
